# Parasitic Diseases, Diagnostic Approaches, and Therapies

**DOI:** 10.1155/2009/893890

**Published:** 2010-01-10

**Authors:** Herbert B. Tanowitz, Louis M. Weiss

**Affiliations:** Albert Einstein College of Medicine, Yeshiva University, Bronx, NY 10461, USA

The diagnosis and treatment of parasitic diseases has undergone major changes because of increased awareness and technological advances that now allow for more rapid and accurate diagnosis of parasitic diseases. These advances are critically important for the continuing diagnosis of these infections as there has been a steady decline in the quantity and quality of laboratory technicians who are expert in the classical techniques of examining stool and blood smears for parasites. The majority of laboratories have an increased reliance on nonclassical parasitological techniques for the accurate diagnosis of these infections. Dr. Ndao from McGill University has given an overview of many of these newer diagnostic methods.

Malaria is a major pathogen in most of the world, and Drs Murray and Bennett from the US Army provide a timely review of the current status of rapid diagnosis of malaria using nontraditional methods. These rapid techniques have been a great advance since, in many laboratories; there are now few individuals that are expert in examining smears. In addition, these rapid techniques can be used by field workers and military personnel. These new methods make the diagnosis more rapid and accurate leading to a more rapid institution of appropriate treatment. Amebiasis continues to be an important cause of morbidity and mortality worldwide. Drs. Singh, Haupt, and Petri, in their review provide an update on the rapid diagnosis of *Entamoeba histolytica*. Diseases caused by Microsporidia are found in both immune-competent and immune-compromised hosts such as those with HIV/AIDS. The diagnosis is often difficult to make. In their article, Drs. Ghosh and Weiss review the state of molecular diagnostics for microsporidian infections. Human infections caused by free-living amoebas have not received sufficient attention in literature despite the fact that they may cause disabilities and death. Dr. Marciano-Cabral's group has reviewed the current state of the diagnosis of these important organisms. The review by Vannier and Krause provides an excellent update on the status of the diagnosis and treatment of babesiosis. Importantly, this infection still poses a threat not only from natural infection via the bite of a tick but also through blood transfusion.

New diagnostic techniques have been developed for metazoan as well as protozoan infections. Neurocysticercosis has received increasing attention as a cause of seizures worldwide. There has also been an awareness of this disease because of the immigration of individuals from endemic areas to non-endemic areas of the world. Drs. Coyle and Tanowitz provide a review of the diagnostic and therapeutic options for management of this infection. Another helmintic disease of humans with complex management issues is that caused by Echinococcus and this is reviewed by Siracusano and colleagues.

The articles by Dr. Baccchi and Dr. de Souza deal with the chemotherapy of trypanosomiasis, both African and American. Drs. Hochman and Kim explore the recent data on the HIV-malaria interaction. Since both HIV and malaria coexist in sub-saharan Africa, this review is timely. Dr. Petersen examines canine leishmaniasis and it implications for human disease.

This year 2009 marks the 100th anniversary of the discovery of Chagas disease. This disease caused by the parasite *Trypanosoma cruzi *continues to be an important cause of cardiomyopathic heart disease in endemic areas of Latin America and is being increasingly recognized in non-endemic areas such as North America and Europe. The article by Dr. Gupta et al. from the Garg group in the University of Texas has offered a unique insight into the role of oxidative stress in the pathogenesis of chagasic cardiomyopathy. Adipose tissue is the largest endocrine organ in the body and its role in infection has only recently been appreciated. The laboratory group at the Albert Einstein College of Medicine offers a review of the role of adipose tissue in the pathogenesis of Chagas diseases, providing a new perspective on this overlooked facet of pathogen host interaction. There has been little success in changing the chronic manifestations of Chagas Disease by using antiparasitic therapy. A new approach to this problem is discussed in the article by Dr. Campos de Carvalho et al. who provide data suggesting that stem cell therapy may be useful in the treatment of the cardiomyopathy caused by *T. cruzi *infection.

We have obtained articles on a range of topics which highlight many of the new issues in the field of parasitological diagnosis and treatment. It is our belief that this collection of articles provides an important summary of these issues and will be of use to both clinicians and researchers working on parasitic diseases. 

*Herbert B. Tanowitz* *Herbert B. Tanowitz* *Louis M. Weiss* *Louis M. Weiss*

